# Protocol Development for Digisection: Making a Case for Standardizing Educational Technology Use for Digital Dissection and Anatomical Studies

**DOI:** 10.7759/cureus.35766

**Published:** 2023-03-04

**Authors:** Joshua Owolabi

**Affiliations:** 1 Anatomy and Neuroscience, Philadelphia College of Osteopathic Medicine, Georgia, USA

**Keywords:** education, medicine, anatomy, protocol, edtech, innovation, dissection

## Abstract

The changes that have characterized advancements in anatomical sciences and medical education have significantly influenced pedagogies and the mode of delivery of teaching and training in the context of medical education. Another reality is the impact of educational technology (EdTech) penetration and integration into medical sciences and education. These events have undoubtedly influenced certain traditional practices and pedagogies including dissection. For example, EdTech and innovations have introduced virtual human images and three-dimensional (3D) human body representations for the purposes of teaching and dissection. Another instance includes the fact that the old dissection guides for gross anatomy, which follow the traditional regional anatomy approach, are often challenging to adapt to the relatively modern medical school curriculum that has embraced the integration philosophy. It is apparent that one practical and realistic way to provide an adapted and effective guide for anatomical dissections under various curricular philosophies and contexts would be to develop *de novo* protocols or adapt existing ones. Protocol development would be a vital component of the modern anatomist toolkit. This article presents the basic considerations and practical approach, including underpinning principles, to developing virtual dissection protocols using a digital 3D dissection facility, the Anatomage table (Anatomage Inc., California, United States of America), as a case study.

## Introduction

Dissection is a practice that is arguably synonymous with anatomy, especially the gross anatomy subdomain. From its root word, it means to cut up for the purpose of exploration, study, or observation. Following this philosophy, anatomical dissection involves methodical, and procedural cutting and exploration of the gross human body for the purpose of appreciating its structural components and correlating such observations with functional, clinical, and applied correlations. Students of anatomical sciences dissect to understand the human body so that they can use such knowledge in applied domains such as clinical practices, surgery, and research [1,2]. *Digisection *is coined from two root words, namely, digital dissection.

Anatomic dissection has been a traditional practice for studying human anatomy for several years. This explains why its strong proponents have argued that it remains a gold standard, which cannot be substituted by any other practice to achieve similar outcomes. On the other hand, the proponents and enthusiasts of educational technology (EdTech) have embraced virtual means of digitally exploring human body structures and functions. In fact, with technologies such as virtual and augmented realities, functional phenomena, and anomalies can be simulated, superimposed, or situated within morphological contexts. An important discourse that has arisen from these realities is whether digital dissection can entirely supplement actual cadaveric dissection. This article is not an attempt to answer this, rather it provides insights into current developments regarding digital dissections as practiced, the need to define what can constitute dissection, and the need for standardization and optimization of such practices. While many anatomists are embracing technology [3], it is clear that ensuring best practices that are also aligned with pedagogical principles has become quite important.

From the root word of dissection, i.e., cut up, digital dissection is feasible as there are educational technologies that represent life-like three-dimensional (3D) images of the human body, enhanced with digital capacities for cutting up and methodically exploring the body. The Anatomage table (Anatomage, Inc., CA, USA) is one such EdTech [4]. While it should be made clear that not all EdTech and innovations are required and designed for digital dissections, the ones that can accomplish digital dissection should meet the criteria of what constitutes dissection. Others can be used as teaching aids and similar modalities including sim-based anatomical education. Digisection is simply used to imply digital or virtual dissection, which is aided by technologies and innovations.

To achieve dissection, the following criteria should be considered: (1) Dissection is a methodical approach to exploring the human body, with specifically defined activities and outcomes. (2) Dissection requires 3D exploration of structures, in relation to surface anatomy, structure relation in situ, and exploration of the structure morphologies without induced limitations. (3) Dissection requires an instructional guide or protocol that defines procedures. (4) Dissection follows a defined pedagogical practice, hence standardized, and not random. (5) Dissection also considers and values applied anatomy and clinical correlations such as variations and aberrations that are observable during activities.

One thing is clear from the above-listed conditions, a standardized practice and protocol guide will be almost indispensable to achieving effective digital dissection. Several authors have generally described various digital approaches to exploring the human body as dissection [4-6]. A word of caution, however, remains that dissection is a methodical and procedural anatomical practice, and not all approaches to exploring human structure beneath the skin might constitute dissection. Clearly, EdTech and innovations can add great value to medical and health science education in the postmodern era. To achieve this, a culture of standard and best practices is required.

Similarly, scholars have proposed virtual dissections with arguments in favor of their benefits [7-9]. It remains noteworthy that dissection is procedural, methodical, scientific, and scholarly; as such, it requires a laboratory protocol or guide to achieve such objectives. Clearly, not all educational use of technology qualifies as dissection in the same manner that not all random exploration of human morphological essence will qualify as dissection. The reason why anatomical dissection can be done in a relatively standardized way with relatively consistent results is its standardization and adherence to a protocol or standardized guide

Prosection basically involves an expert pre-dissecting the human body for learners and trainees to obverse or use for anatomical studies. In a similar way, several EdTech and innovations allow for pre-dissected human body parts and organs to be visualized and studied. In addition, these EdTech and innovations can allow for functional observations while exploring or dissecting, thus helping learners to appreciate functional phenomena, such as valvular closure and reopening during rhythmic heart contractions, pregnancy stages and accompanying developmental features, nerve impulse conductions, and blood flow through vessels.

## Technical report

Digital dissection: basic pedagogical principles and considerations

The use of EdTech and innovations should align with pedagogical principles. The following considerations would be quite important [10]:

Consideration 1: Provide protocol details. Every protocol should have general details that include the session title, course title, dates, and identity such as the protocol number.

Consideration 2: Define the activities. Provide session titles, an introduction, and assigned pre-session study materials if needed.

Consideration 3: Indicate the session’s objectives. State specifically the session’s objective or outcomes. A set of four to eight clear statements that define the expected outcomes would be quite helpful.

Consideration 4: Define the target students. Indicate the group of students by clearly naming the class or student cohorts.

Consideration 5: Time allocation. Indicate the time allocated for the activity, preferably in hours or minutes. This might also include the time block (e.g., 4:00-6:00 pm).

Consideration 6: Learners’ attributes. Consider learners’ attributes in determining the workload and the level of assessment. Bloom’s taxonomy and Miller’s pyramid can be helpful guides. Though not required, clarifications can be provided by indicating whether the session is focused on basic medical science knowledge, clinical skills lab, or a simulation session.

Consideration 7: Pedagogical approach. Determine clearly the pedagogical approach, whether this should be a dissection, prosection, or simulation session.

Consideration 8: Activities and procedures. Determine, outline, and organize activities that constitute the session into a logical procedure that can be conducted in the most effectively procedural method.

Consideration 9: Assessment of learning (consider Miller’s pyramid). Determine the most appropriate assessment methods and prepare the assessment including how the assessment will be graded.

Consideration 10: Feedback, debriefing, and evaluation. Plan debrief sessions, learners’ feedback, and the most appropriate measures of tracking performances and evaluating activities for improvement.

Protocol template: basic elements

It is important to consider the ideal elements that constitute a quality protocol template. Clearly, the activities or procedures are the most important components of the template document. Nevertheless, there are other important elements to ensure that users have optimal experiences and proper guidance. While the list below is not exhaustive, it can be quite helpful [10].

Protocol template basic element 1: General protocol information. This might include the session title: MED (Medicine) 204; laboratory name, e.g., Anatomage Lab (Laboratory) Session I; activity venue, e.g., Lab 2; faculty/instructor’s name, e.g., Dr. Alex Adams, etc.

Protocol template basic element 2: General introduction and laboratory description. This part is typically a short paragraph that addresses what the session is all about.

Protocol template basic element 3: Brief review of subject and scientific principles. It can typically include a short knowledge-based note to connect practical or hands-on session activities with the base knowledge or information that projects the session unto application activities.

Protocol template basic element 4: Learning objectives. This is a very important and indispensable component of a protocol. Every session should have clearly formed objectives or outcomes. Typically, four to eight is the optimal range.

Protocol template basic element 5: Safety Notes. For an EdTech or device, it is key to provide safety notes for users, especially when dangers such as electric shock or physical traumas might be involved. There should be caution notes on how to prevent damage to devices as well.

Protocol template basic element 6: Materials. If there are other materials and resources that you have provided for enhancing students’ learning experiences, such as models and tools, please indicate or list them and state how they can be used.

Protocol template basic element 7: Detailed protocol. This part contains the session’s set of activities. Note that dissection is practically procedural, implying that the activities consist of a series of actions that are performed in a logical and practical order that leads to accomplishing the session’s objectives/outcomes. An important thing to do is to link activities to outcomes and arrange them in a practical order. It should be clear how these activities will lead to the accomplishment of the outcomes or objectives. This is the core aspect of the protocol.

Protocol template basic element 8: Description of assessment. Information on assessment is required (if there would be an assessment during/after the activity). This might include a short paragraph with a link for online assessment or information on what to do on a paper workbook/sheet. This should also include submission information.

Protocol template basic element 9: References. If applicable, references should be included for further studies or consultation. This can include atlases, texts, web pages, media, etc. It should be for revision or consultations.

Protocol template basic element 10: Approval. It is important to include whose approval has been obtained for the use of the protocol. Typically, this might include the lab manager, the faculty in charge, and the chair of the division. This helps to validate the document.

Protocol template basic element 11: Appendices. These are other documents that complement the session’s activities. These might be consulted by students during sessions, such as technology user guide, sample work, reference images, etc. If applicable, the exercise and workbook document should also be provided as an appendix.

Protocol template basic element 12: Others. You may include post-class activities or links to supporting documents as applicable.

Protocol use and EdTech optimization: ASIC (adaptation, standardization, integration, compliance)

It is important to appreciate the fact that adherence to relevant educational and pedagogical principles would contribute significantly to optimizing EdTech and innovations’ benefits when used to support anatomical and medical education. To this end, the ASIC framework has been promoted to serve such purposes [11-13]. It has four tenets, including adaptation, standardization, integration, and compliance. In its original publication, the ASIC framework emphasizes and illustrates the need to assure the four aforementioned tenets have also been presented in the form of an operationalizing matrix. The fact that the ASIC framework can be adapted to different educational contexts makes it a suitable framework for anatomists and medical educators globally. In addition to this is the fact that it remains a foremost framework for adapting, standardizing, and integrating EdTech with an emphasis on the need to assure compliance with institutional culture, structure and practices, regulatory policies and requirements, and pedagogical principles.

Benefits of protocol development

Laboratories sessions are practical activities or skill sessions typically requiring a protocol or guide. Digisection, being practical, should also require protocols, guides, or laboratory manuals [14,15]. Some of the key benefits of using protocols are listed below.

Problem statement 1: EdTech use often does not comply with standard anatomical practices. The primary requirement or condition to address this is standardization. Protocol development and use to deploy EdTech assure standardization.

Problem statement 2: Many curricula have no indicators of assessment of competencies with EdTech. The principal requirement is to assure adaptation. Protocol development assures critical appraisal of activities in alignment with competencies, as indicated in program documents.

Problem statement 3: The use of EdTech, even of the same types, can greatly vary from place to place and can depend on interest, technical competencies, context, and systemic factors. With EdTech, there is ample latitude for creative use and creative adaptations based on factors such as level of technological appreciation, competencies, and systemic factors. Protocol development helps with the standardized use of EdTech and innovations, as well as replicability to ensure consistency in use.

Problem statement 4*: *EdTech is rapidly proliferating, and educators and regulators find it difficult to standardize them all. It might be impractical to ensure that all educators would use EdTech in the same way; diversities exist in curricular structures, program design, infrastructural support, and institutional culture. However, development and implementation protocols in line with anatomical pedagogical principles and practices will assure quality adaptations, standardization, and best practices.

Problem statement 5: EdTech use is not necessarily standardized by makers. EdTech makers have educators and technology experts that participate in design, production, and training-the-trainer programs. Most standard EdTech and innovations meet minimal standards to serve their purposes. Governments and regulators set for them the minimal standards to which they adhere. However, in line with tech culture, the specific use of EdTech will require the requisite skill sets and appreciation of educational theories and pedagogical principles as well as standard practices to optimize their use. This is why a protocol is required to assure such optimization.

Problem statement 6: EdTech is rapidly proliferating, causing heterogeneity. The use of protocols will help to assure the standardized use of EdTech and innovations, as well as consistencies in such practices. In addition, protocols will assure adherence to pedagogical principles and ASIC tenets as highlighted.

Problem statement 7: Regulators are often averse to EdTech use. Regulators often value evidence-based practices, and they value adherence to standards and policies. Protocol development validates evidence use in educational practices and feedback that can improve regulatory policies and required standards.

**Figure 1:**
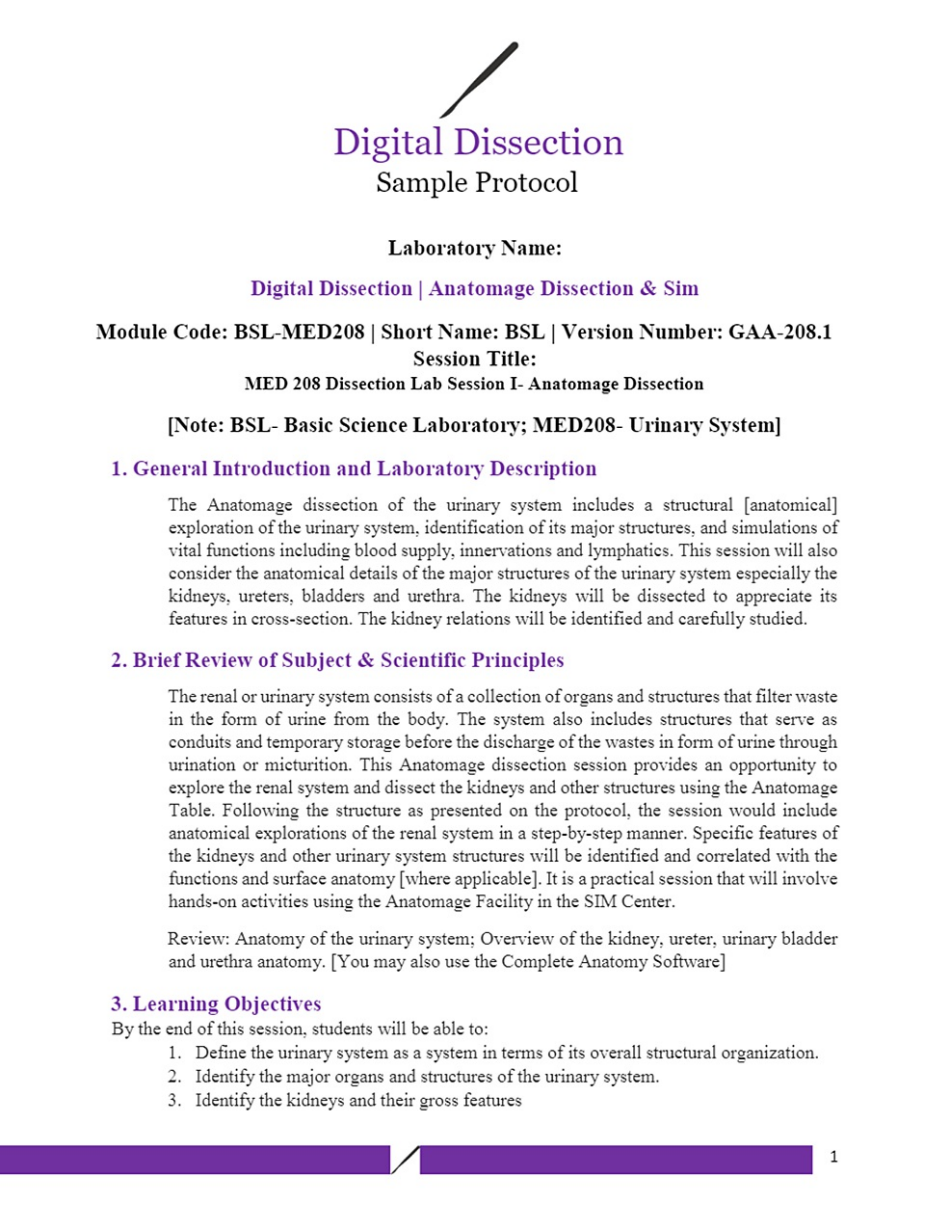
Digital dissection sample protocol template: page 1. BSL: Basic Science Laboratory; MED: Medicine; MED 208: Medicine 208 Course; GAA 208.1: Gross Anatomy Dissection Using Anatomage in the Medicine 208 Course, Session 1; MED 208: Course title is The Urinary System

**Figure 2:**
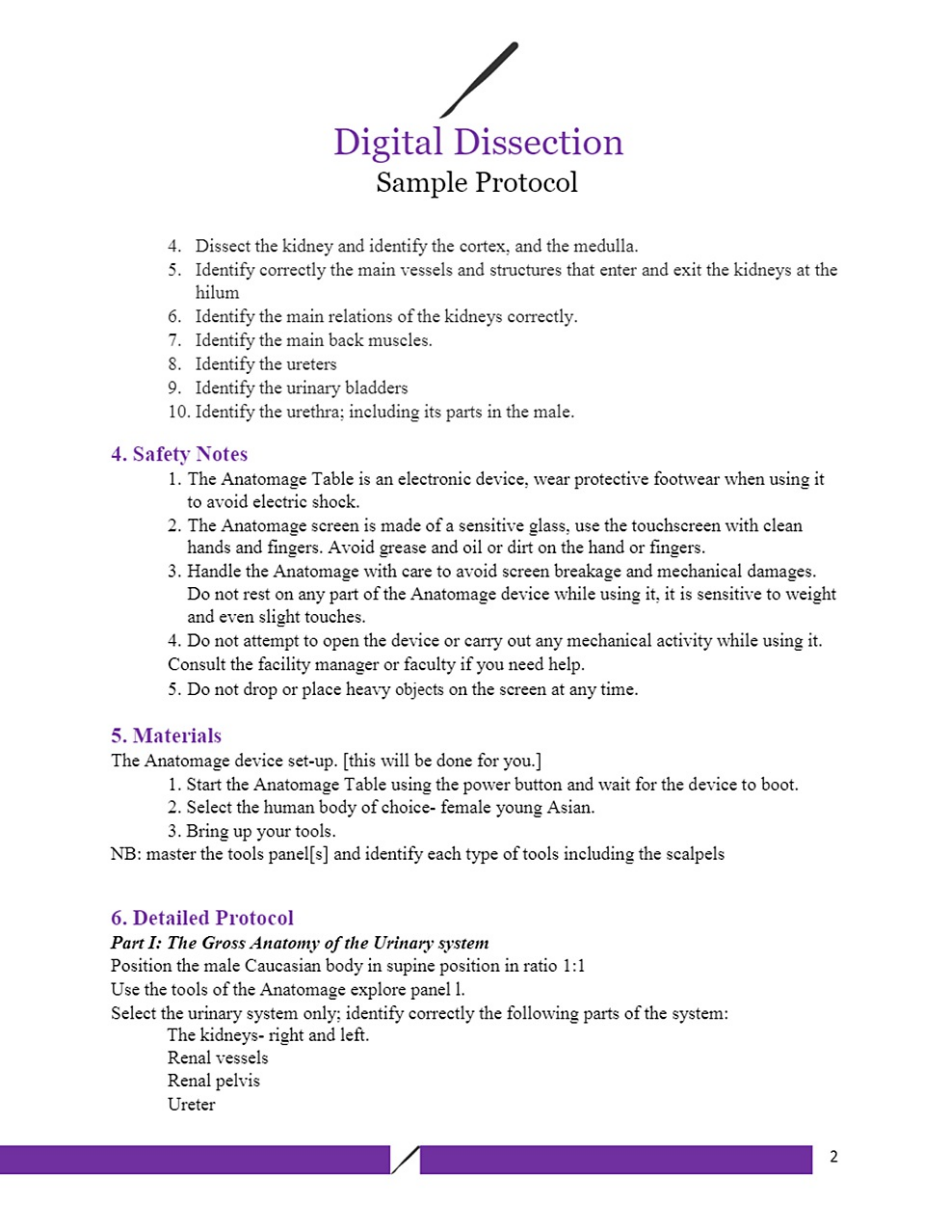
Digital dissection sample protocol template: page 2. BSL: Basic Science Laboratory; MED: Medicine; MED 208: Medicine 208 Course; GAA 208.1: Gross Anatomy Dissection Using Anatomage in the Medicine 208 Course, Session 1; MED 208: Course title is The Urinary System

**Figure 3:**
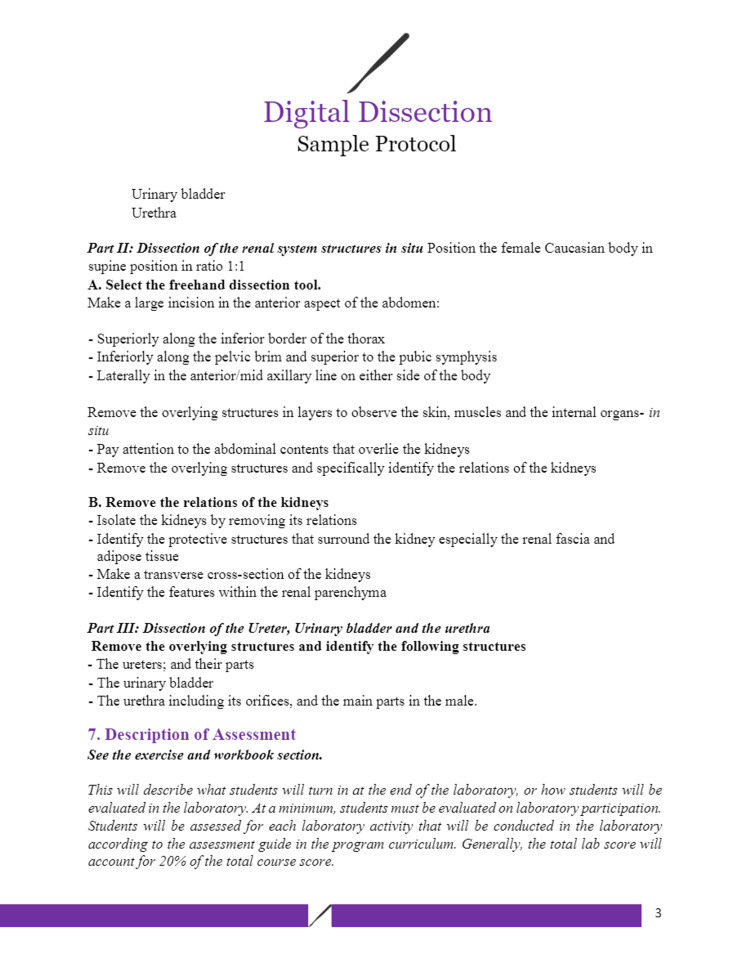
Digital dissection sample protocol template: page 3. BSL: Basic Science Laboratory; MED: Medicine; MED 208: Medicine 208 Course; GAA 208.1: Gross Anatomy Dissection Using Anatomage in the Medicine 208 Course, Session 1; MED 208: Course title is The Urinary System

**Figure 4:**
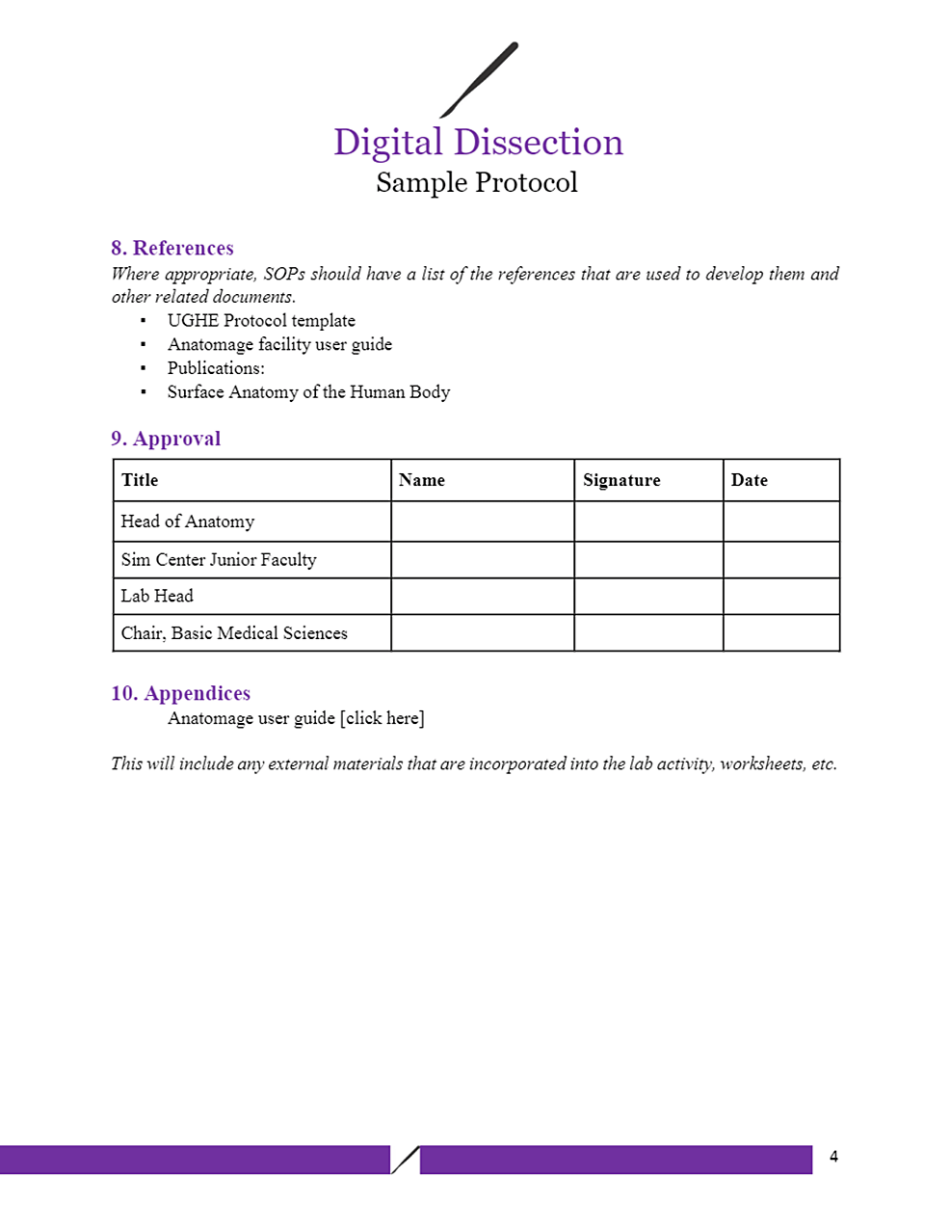
Digital dissection sample protocol template: page 4. BSL: Basic Science Laboratory; MED: Medicine; MED 208: Medicine 208 Course; GAA 208.1: Gross Anatomy Dissection Using Anatomage in the Medicine 208 Course, Session 1; MED 208: Course title is The Urinary System

**Figure 5:**
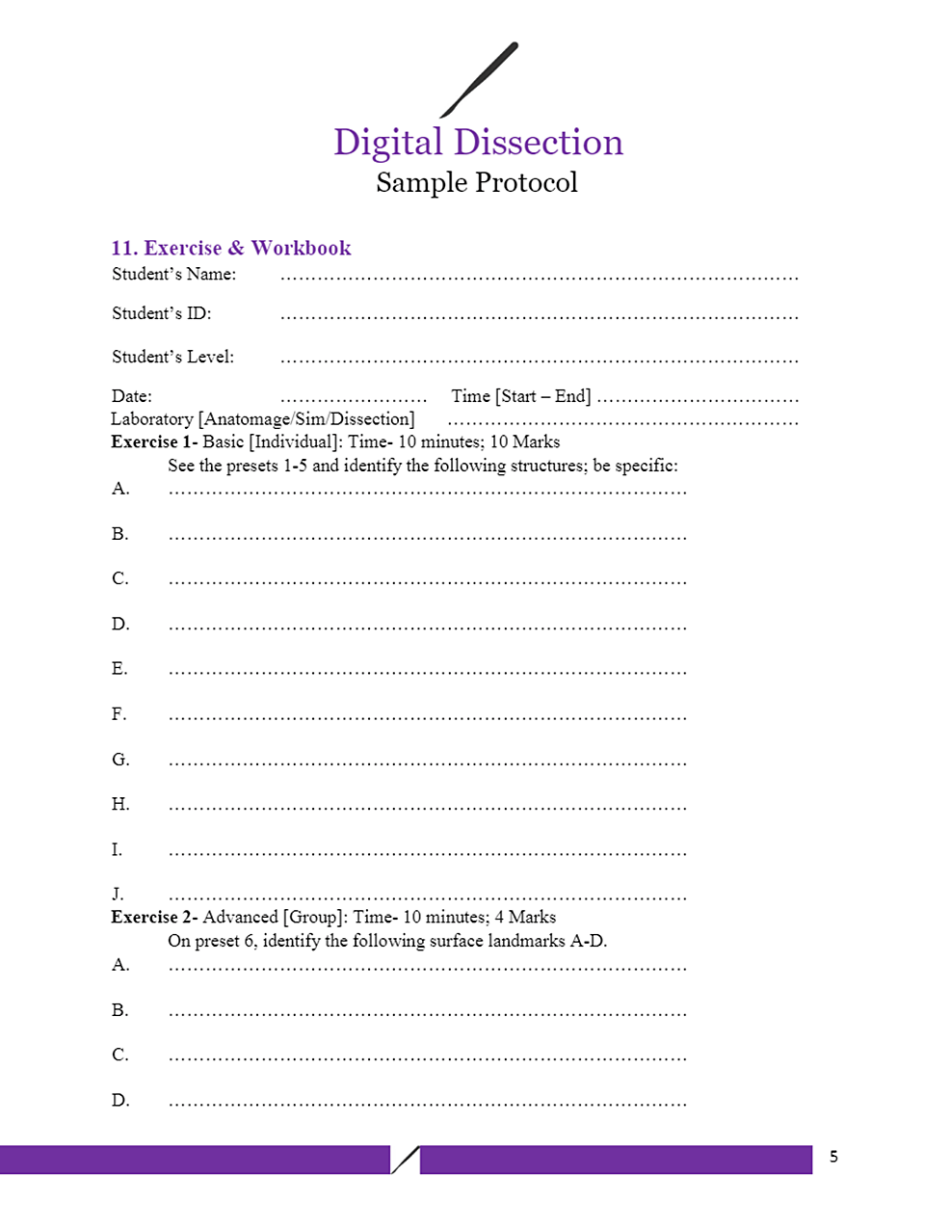
Digital dissection sample protocol template: page 5. BSL: Basic Science Laboratory; MED: Medicine; MED 208: Medicine 208 Course; GAA 208.1: Gross Anatomy Dissection Using Anatomage in the Medicine 208 Course, Session 1; MED 208: Course title is The Urinary System

**Figure 6:**
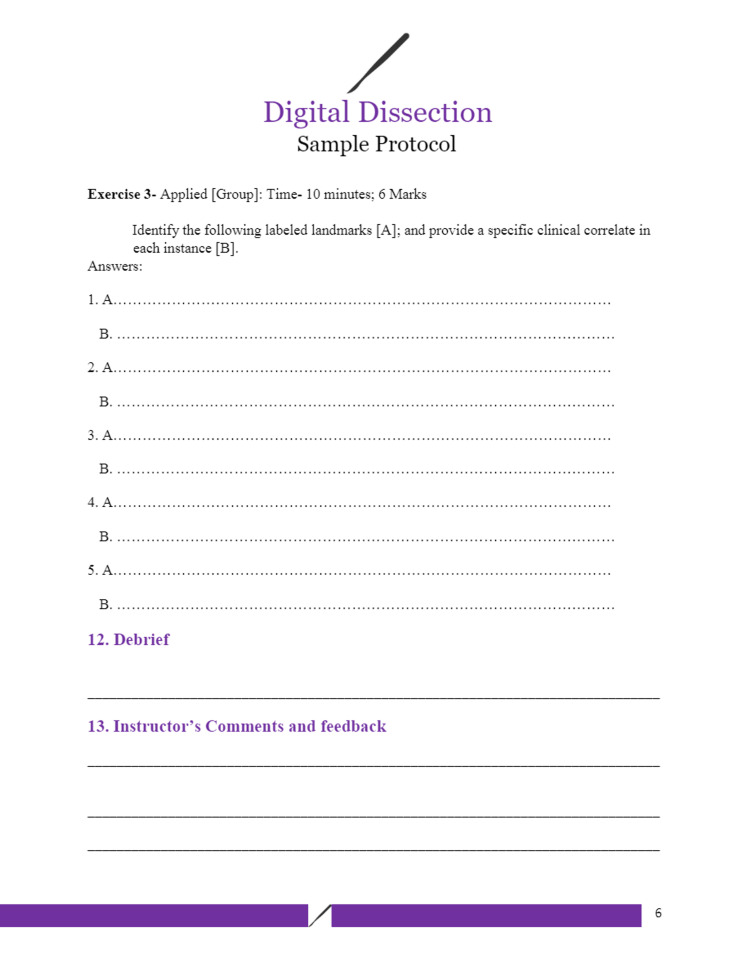
Digital dissection sample protocol template: page 6. BSL: Basic Science Laboratory; MED: Medicine; MED 208: Medicine 208 Course; GAA 208.1: Gross Anatomy Dissection Using Anatomage in the Medicine 208 Course, Session 1; MED 208: Course title is The Urinary System

Figures 1-6 include pages 1-6 of the digital dissection sample protocol.

## Discussion

Medical education and anatomical science in recent times have witnessed significant advancements, not just in curricular content but also pedagogies and methods of training. Factors that have contributed to these advancements include innovations and educational technologies. Moreover, new variants of curricula have emerged, and these have also necessitated adaptations in methods of teaching and training. The variants of curriculum that have emerged have significantly influenced anatomical dissections. For instance, while the traditional dissection methods and approaches favor regional anatomy, the relatively newer curricula favor functional integration of anatomy with other disciplines, which is largely system-based. Technology clearly has roles to play in efforts to adapt to these changes [16]. A major advantage of EdTech and innovation is dexterity regarding adaptions and applications.

Another important development is the introduction of innovations and EdTech into the teaching of anatomical sciences and medical education in general. The challenge that has come with this, in this context, is the heterogeneity that has characterized the pattern and levels of innovations and EdTech penetration and integration. These might have resulted from significant variations or divergences in perspectives, philosophy, and skills to use EdTech, especially by educators and trainers. There have been calls to address this reality of heterogeneity to standardize EdTech use for the benefit of learners. To this end, a crucial way to address the typical lack of congruence between most traditionally available dissection guides and the mode of delivery of anatomical teaching in the context of many modern curricula is to equip anatomists with protocol development and implementation skills, such that they can either adapt existing protocols or develop new protocols for anatomical dissections, irrespective of curricular variant and context. It would, therefore, suffice to say that protocol development and implementation have become requisite skills for the modern anatomist. The roles of technologies in anatomy teaching have been demonstrated or advocated for [16-18]. Assuring standard and best practices remains a goal to achieve.

Another vital consideration in this regard is the need to standardize the use of EdTech and innovations for practical anatomy sessions with special emphasis on digital or virtual dissection. Consideration for *balance* regarding EdTech use has been previously highlighted [16]. This is an important way to ensure that the use of EdTech and innovations are in line with curricular requirements and competencies that constitute program and training outcomes. This article is, therefore, a reflection, technical report, and an experiential piece that presents the basic considerations of protocol development guiding principles, a typical template, assessment method, as well as means of evaluating protocols for further improvement and system strengthening. To illustrate digital dissection protocol development principles and practical methods, the Anatomage table, which is the foremost anatomical EdTech, is being used as a case study.

Furthermore, it is important to emphasize that protocols are typically required in teaching laboratories and facilities, especially in the basic medical sciences domains. This is to ensure that students learn procedures and processes and that they can be assessed against such standard practices [19]. The perceptions and positions of anatomists regarding dissection and related practices are evolving, yet without a universal consensus [20]. Efforts should rather be invested in assuring best and standard practices. There is a need to benefit from the creative edge that technologies offer when properly deployed to support the teaching of anatomy or other related basic medical sciences. However, this creative edge must not be in abeyance to sound scientific and pedagogical principles upon which educational practices are fundamentally premised.

With technologies, the level of integration and effectiveness of use often depends on the level of technical competence, enthusiasm, and technical skills of the educator. A tech-savvy educator might be able to use a particular EdTech much more effectively and robustly in comparison with another who is tech-averse or whose interest in EdTech is minimal. Such human factors can make significant differences in the impacts of EdTech use even when similar EdTech or innovations are deployed. In fact, an overly enthusiastic user of EdTech and innovations but with limited technical skills and appreciation for pedagogical principles regarding technologies might misuse EdTech, causing abuse or misapplication relative to the anticipated training outcomes. This explains why there should be a consistent emphasis on the need to assure optimal deployment of EdTech and educational innovations through protocol development and use. Because with EdTech, it is impractical to expect each innovation or technology to come up with a protocol for its use for teaching and training, there is a need for educators or anatomists to develop protocols, student guides, or manuals.

## Conclusions

EdTech and educational innovations have immense value to contribute to the advancement of anatomical and medical education. It is also relevant to anatomical dissection, which can be done digitally or virtually using suitable educational innovations or EdTech. The need to use EdTech in standardized ways and following best practices requires the use of guides and frameworks, the purpose of which protocols typically and traditionally serve. This article has made a case for the need to develop and use protocols to assure the optimization of EdTech and innovations for teaching anatomical sciences in the form of digital and virtual dissections. In addition, it makes a case for a need to ensure that anatomists who use EdTech have protocol development and implementation skills to ensure optimal deployments and benefits that come with EdTech use to support anatomy teaching. It is important to note that further studies on EdTech and innovation use for anatomical science education to obtain empirical data toward establishing the reliability and validity of digisection protocols and guidelines would be quite helpful.
